# A feasibility study of a mobile phone supported family-centred ADL intervention, F@ce™, after stroke in Uganda

**DOI:** 10.1186/s12992-018-0400-7

**Published:** 2018-08-15

**Authors:** Julius T. Kamwesiga, Gunilla M. Eriksson, Kerstin Tham, Uno Fors, Ali Ndiwalana, Lena von Koch, Susanne Guidetti

**Affiliations:** 1 Division of Occupational therapy, Department of Neurobiology Care Sciences and Society, Karolinska Institutet, Stockholm, Sweden; 2 Uganda Allied Health Examinations Board, Kampala, Uganda; 3 Department of Neurology, Karolinska University Hospital, Stockholm, Sweden; 4 Department of Neuroscience, Rehabilitation medicine, Uppsala University, Uppsala, Sweden; 5 Department of Computer and Systems Sciences (DSV), Stockholm University, Stockholm, Sweden; 6 Malmö University, Malmö, Sweden; 7 Knowledge Consulting Ltd, Kampala, Uganda

**Keywords:** Tele health, Africa, ICT, Low-income, Occupational therapy, SMS, Telerehabilitation, Tele medicine, Stroke rehabilitation, Participation

## Abstract

**Background:**

There is a lack of evidence-based health services to reduce the impact of stroke in low-income countries at a personal, family or community level.

The aim was to evaluate the feasibility of: i) a mobile phone supported family-centred intervention (F@ce™)*,* and ii) the study design for evaluating the effects of the intervention on the perceived impact of stroke; perceived participation in everyday life; and self-efficacy in everyday activities amongst persons with stroke and their families in Uganda.

**Methods:**

The study comprised a pre-post design with an intervention group (IG) receiving the F@ce™ and a control group (CG). The inclusion criteria’s were: a) confirmed stroke diagnosis, b) access to and ability to use a mobile phone, c) ability to communicate in English and/or Luganda, d) > 18 years, e) residents in Kampala, and f) a Modified Rankin Scale level 2 to 4.

The aim of the F@ce^TM^ was to increase functioning in daily activities for persons living with the consequences of stroke, and participation in everyday life for persons with stroke and their families. The F@ce™ was an eight-week family-centred intervention, which entailed goal setting and problem-solving strategies, daily reminders and self-rated follow-ups of performance by short message service (SMS).

Data were collected in the participants’ home environment at baseline and after eight weeks. Data on acceptability of the F@ce™ and study procedures were collected by log-books and the responses of the SMS follow ups on the server. The primary outcomes were performance and satisfaction of valued daily activities in everyday life using the Canadian Occupational Performance Measure (COPM), self-efficacy in performance of activities in daily life.

**Results:**

The IG comprised *n* = 13 and the CG *n* = 15. There were differences between the IG and CG in changes between baseline and follow-up in the primary outcomes COPM (performance component) and self-efficacy in favour of F@ce™. Overall with minor modifications the intervention and the study design were feasible for all participants involved.

**Conclusion:**

The results support the need for further research to rigorously evaluate the effects of F@ce™ since the intervention appears to be feasible for persons with stroke and their family members.

**Electronic supplementary material:**

The online version of this article (10.1186/s12992-018-0400-7) contains supplementary material, which is available to authorized users.

## Background

Stroke is one of the non-communicable diseases that pose a global challenge. Fifteen million people suffer a stroke annually, and one third of them will subsequently live with disabilities [1]. The global burden of stroke is increasing, and there is lack of evidence of how to lessen its effect at a personal, family or community level. The burden of stroke in Africa is rising substantially [2, 3], and stroke causes impairment, activity limitations and restricts participation potentially resulting in decreased functioning in everyday life [4]. Furthermore, the World Health Organization estimates that stroke currently ranks as the number five cause of mortality in Uganda [5]. Stroke can lead to a stressful situation for family members, with restricted participation, reduced life satisfaction and perceived burden among caregivers [6, 7]. A general aim for most rehabilitation programmes for people with stroke is, therefore, to involve the persons’ family in order to attain maximum participation in activities in daily living (ADL) [8]. Contemporary theories have been confirmed in empirical studies in rehabilitation after stroke, and have shown strong evidence of positive effects of ADL interventions [9, 10]. Therefore, activities that are relevant and purposeful for people in everyday life can be used as targets to improve ADL functioning [11, 12].

Most research and evidence on beneficial rehabilitation interventions after stroke originates from high-income countries, but evidence is lacking that such interventions can be implemented with similar outcomes in the context of sub-Saharan Africa. Uganda, like any other low-income country, has limited rehabilitation services due to poor infrastructure, inadequate numbers of rehabilitation professionals, and poor health support systems in addition to the poor socio-economic situation of the people.

In 2013, the World Bank estimated that 34.6% of Uganda’s population was living below the extreme poverty line, on $1.90 per day [13]. Furthermore, the majority of the Ugandan population (84%) then lived in rural areas where medical rehabilitation is almost non-existent [14]. To increase the accessibility and affordability of rehabilitation services to those that are in dire need, alternative approaches to providing rehabilitation is urgently needed.

Telerehabilitation could offer opportunities to provide cost-effective rehabilitation services, and potentially resolve some unmet needs of rehabilitation, by utilization of information and communications technologies (ICT), to facilitate health and wellness. The use of ICT could provide health services for individuals with disabilities living in rural communities at some considerable distances from the rehabilitation provider [15, 16]. Healthcare professionals and participants have reported high levels of satisfaction and acceptance of telerehabilitation interventions in stroke care, although few studies have explored this outcome extensively [17]. This could also alleviate rehabilitation workforce shortages, as more people with stroke can receive therapy services without generating a severely increased financial burden [15].

A systematic review showed that the use of telerehabilitation after stroke offers a wide range of treatments, but whether telerehabilitation is an effective way to provide rehabilitation was inconclusive [18]. Another review provided only limited, moderate evidence that telerehabilitation is almost as effective as conventional rehabilitation in improving ADL abilities and motor function for stroke survivors [19]. Additional trials in the field of rehabilitation are urgently recommended to extend the evidence-based knowledge [18, 19]. Studies are needed on the use of telehealth technologies in professional development and supervision to clarify effectiveness and efficiency as an urgent demand for services, particularly in rural areas, threatens to exceed the actual services currently available [15].

Mobile phones have been rapidly integrated into everyday life in countries in East Africa [20]. At the end of 2016, there were 420 million unique mobile subscribers in sub-Saharan Africa, equivalent to a penetration rate of 43% (https://www.gsmaintelligence.com). According to the GSMA Intelligence Global Mobile Engagement Index (GMEI) report 2017, sub-Saharan Africa will reach even higher levels of mobile engagement in the coming years, underpinned by growing access to mobile data services and smart devices.

A previous study from Uganda [21] showed that the mobile phone was experienced by persons who had had a stroke and their families as an important device that could facilitate change and promote ADL functioning after stroke. The mobile phones appeared to be an accessible and affordable technology used in the daily life of persons with stroke and family members, and connected them to the services and social relationships that were needed [20].

Following the Medical Research Council (MRC) guidance for developing and evaluating complex interventions, [22] a client-centred activities of daily living (CADL) intervention was developed and evaluated in Sweden [11, 23]. The CADL intervention applied a client-centred approach by taking the person’s lived experiences as the point of departure for collaboration and goal-setting during the intervention process [11, 23]. To apply CADL in Uganda, an adaptation of the Swedish version was required, since the health support system in this context is mostly provided by the person’s own family.

In this feasibility study, the intervention F@ce™, which uses a mobile phone to support the intervention, was evaluated together with the study design. In F@ce™, F stands for Face-to-face between the therapist and a client, @ for Assessment, C for Collaboration and E for Evaluation. A feasibility study is intended to estimate essential parameters required to design a full-scale study, such as sample size, outcome measure, recruitment of participants, response rates and adherence, availability of professionals to carry out interventions, follow-up rates, etc. [24]. The aim of this study was to evaluate the feasibility of: i) a mobile phone supported family-centred ADL intervention F@ce™*,* and ii) the study design for evaluating the effects of the intervention on the perceived impact of stroke, perceived participation in everyday life, and self-efficacy in everyday activities among persons with stroke and their families in Uganda.

## Methods

### Trial design

This study was conducted using a pre-post design with an intervention group (IG) receiving the F@ce™ intervention and a control group (CG). The CONSORT statement for non-pharmacological trials [25, 26] was used as the frame of reference.

### Participants

The inclusion criteria for participants with stroke were: a) a stroke diagnosis (haemorrhage/infarction/an unspecified) confirmed by computerized tomography (CT) and/or clinical signs, b) access to and ability to use a mobile phone, c) ability to speak and express themselves in English and/or Luganda, d) > 18 years of age, e) resident in Kampala and its surroundings < 40 km, and f) a Modified Rankin Scale (mRS) [27] level 2 to 4. The mRs levels = 0) no symptoms, 1) no significant disability, despite symptoms – able to perform all usual duties and activities, 2) slight disability – unable to perform all previous activities but able to look after own affairs without assistance, 3) moderate disability – requires some help, but able to walk without assistance, 4) moderately severe disability – unable to walk without assistance and unable to attend to own bodily needs without assistance, 5) severe disability – bedridden, incontinent, and requires constant nursing care and attention, 6) dead [27, 28].

The inclusion criteria for the family member were: a) the family member who mainly helped and lived together with the person with stroke, and b) was able to understand and respond to instructions in English or Luganda. The participants with stroke and family members were informed about the study, after which an informed consent was signed.

### Recruitment and data collection

Persons with stroke who met the inclusion criteria and one of the family members were recruited between March 2015 to March 2016 from four different sites in Kampala, Uganda: a) the Neurology ward of Mulago Referral Hospital, b) the Physiotherapy outpatient clinic at Mulago Referral Hospital, c) the Stroke Rehabilitation Centre, d) Nagulu Hospital, and e) referrals from colleagues of the first author (JTK). These were the main healthcare units in Kampala and surrounding where people with stroke could be admitted for acute stroke care and/or stroke rehabilitation. Mulago hospital is the only public general referral hospital serving Kampala city with the population of 1.5 million people and the entire country with estimated population of 40 Million. Transportation is complicated due to the poor roads, heavy traffic jams and the poverty amongst the people. The group allocations were conducted by JTK. The first participant, who met the inclusion criteria and agreed to participate in the study was randomized by flipping a coin and allocated to the IG. The next participant was allocated to the CG. Thereafter, every other participant was enrolled to the IG and the CG, respectively.

The staff at the recruitment sites reported to JTK when they identified a potential client who met the inclusion criteria. The first meeting was held in the hospital or physiotherapy clinic where screening of the clients was conducted by JTK using the inclusion criteria. The client was informed orally about the study, and asked if he or she agreed to participate, and was then asked to sign a written consent. An appointment was made after a week to two months depending on whether the client was first met in the acute neurology ward or in the physiotherapy rehabilitation clinic. The researcher and one of the occupational therapists (OTs) was assigned to give the intervention and met the client in their home environment. During the home visit, the baseline data were collected. The participants allocated to the CG were assessed using the same assessment protocols as those used for the IG. The protocols, including demographics and assessment instruments, were installed by an ICT consultant in Uganda with access to the local network on a tablet in an application called Open Data Kit (ODK) collect (https://opendatakit.org/about/full). This was intended to make data collection easier, more secure and more accurate, as data were uploaded and stored to a cloud immediately, and it was not possible to skip items unintentionally. All data such as participants’ names and mobile phone numbers were unidentified and encoded with serial numbers. JTK was involved only during the baseline and follow-up assessments, and did not take part in delivering the F@ce™ intervention. At the time of the study, it was not possible to find another person qualified to collect the data, hence there was no blinding or allocation concealment.

### Sample size

In this feasibility study, a prospective sample size was not calculated. A sample with more participants would provide greater precision of scores for the outcomes [29], but there is no definitive sample size recommended for feasibility studies, rather a range from 10 to 50 participants or more. A total sample size of 30 individuals, 15 in each group, was deemed to be viable.

### Planning and modelling the intervention

The development of the F@ce™ intervention in Uganda was initiated after conducting a focus group interview with OTs and physiotherapists in 2014 which explored the possibility of using mobile phones for rehabilitation in the Ugandan context. Subsequently, a series of meetings and workshops for modelling the components in the CADL intervention took place both in Sweden and Uganda. The modelling of the Ugandan intervention was tailored to local preferences and expressions in order to achieve a good fit with existing practices and values, e.g. family-centred intervention, cost-free, use of participants’ own ordinary phones, easily accessible, low cost, and likely to be sustainable. A core component in the intervention was to set goals together with the clients, and semantic adaptation was strongly advocated by the participating OTs and instead setting targets was used.

Thereafter, eight half days of training workshops were held for the five local OTs who were to deliver the mobile phone-supported family-centred ADL intervention (F@ce™). The OTs all had work experience of not less than two years. The overarching goal of the workshops was to facilitate the implementation process of the F@ce™ intervention for the OTs who participated.

### The F@ce intervention

The F@ce™ intervention was an eight-week intervention which applied a phenomenological perspective using the participants’ lived experiences as the point of departure for the family-centred intervention, i.e. the training/practice was adjusted to the individual participant’s ability, motivation, perceptions and needs, and in close collaboration with the family members during the entire intervention process. The aim of the intervention was to increase the functioning in daily activities for persons living with the consequences of stroke, as well as participation in everyday life for persons with stroke and their family members.

*Guiding principles used in F@ce are presented in* Additional file 1*:* Appendix 1*.*

The participant was introduced to a problem-solving strategy framed as Target-Plan-Perform-Prove strategy, intended to facilitate the learning and problem-solving process to be used during the intervention. The strategy also provided a structure for the OT, the participant and the family member to benchmark the problems encountered in the performance of the target activities [30]. The Canadian Occupational Performance Measure (COPM) [31, 32] was then used by JTK and the OT that together with the participant formulated three targets in daily activities that the person wanted and needed to do, and that were measurable and feasible to the level of his or her abilities within the home environment. Each activity was practiced by the person who had had a stroke together with the OT and the family member, to discover and identify difficulties in performance of the activities chosen as targets.

Different strategies were used, such as finding new ways to perform the activities and/or modifying the environmental demands to enable performance.

JTK informed the other family members about the participant’s target activities and the planned strategies for reaching these targets. To recapture the target activities, the participant was told to look at the sheet with the formulated targets every morning, and then practice the chosen activities.

### The use of the mobile phone in the implementation of F@ce™

The participants received individual short message service (SMS) on their mobile phones containing the three targets twice daily, morning and evening. The morning message was to remind the participant to perform the activities during the day. The participants were to practice the target activities in their home environment. In the evening, the participant, with or without support from the family member, was to respond in three separate SMS, containing the daily performance scores, one for each target activity. A five-point rating scale on a prepared sheet of paper was left with the participants to rate their own performance of the three target activities for replying to the SMS, where 0 meant “has not performed the activity” and 5 meant – “carried out the activity well”. Participants who rated 0 or who did not reply to the SMS reminder, automatically launched a red flag on the OT’s mobile phone. The OT would call the participant the following morning to find out what had happened. When the client could not manage the mobile phone, the family member was to receive the SMS, support the client to rate the performance, send scores by SMS and encourage the client to perform the activities. Additionally, the participants were to receive mobile phone calls from their OT twice a week during the intervention. The calls were a follow-up strategy aimed at exploring and resolving issues that might have affected achievement of the targets. When a target was reached the instruction was that during the scheduled phone call, the participant and the OT should discuss and evaluate the strategies implemented and then, together, formulate a new target.

JTK supplied the participants with mobile phone airtime daily to enable them to send their replies on the SMS system free of charge. All the participants’ data, targets and SMS messages went back and forth to a server based in Sweden and were handled by the server-based SMS service developed by the research team.

### Intervention group (IG)

During the home visit and after the assessment, the plan for strategies used to reach targets was set with the person with stroke and their family member. The IG received the F@ce™ intervention.

### Control group (CG)

Participants in the CG did not receive any rehabilitation supported with SMS messages as in the F@ce™ intervention.

### Both groups

In both groups, the researcher gave information about stroke during the initial assessments, and advice to promote independent functioning in ADL. Also, the participant’s blood pressure (BP) was measured using a digital BP machine, and balls for training hand strength were handed out to all participants who had impaired hand motor function. Participants, both in the IG and the CG, could receive other rehabilitation services such as physiotherapy and speech therapy.

### Demographic data and clinical characteristics

Demographic data were collected at the onset for both the IG and CG related to age, gender, civil status, living conditions and level of education. At baseline, the Scandinavian Stroke Scale (SSS) was used to describe the clinical characteristics, assess neurological impairment and define stroke severity [33–35]. SSS is designed to give a score based on the level of consciousness, eye movement, orientation, speech, hand and leg movement, gait, and facial paralysis. The SSS has been used extensively in clinical trials and has been shown to have high inter-observer reliability (0.93) and high concurrent validity (0.94–0.98), especially when performed face-to-face [36]. The SSS scores ranges from 0 to 58 points, where 0 is severe neurological impairment.

### Outcome measures

#### Feasibility of the intervention

To evaluate adherence to the F@ce™ intervention, the OTs kept logbooks about their follow-up calls, and all other services related to the intervention.

Further, the log-books recorded the feasibility of the estimated parameters; sample size, recruitment of participants, response rates, adherence and follow-up rates as well as the possibility and acceptability of OTs to carry out F@ce ™. Additionally, the feasibility of the selected outcome measures were based on their capacity to detect changes between baseline and follow-up after the intervention phase.

#### Primary outcome measures of the intervention

The primary outcome measures comprised the COPM [37], Self-efficacy [38, 39], and the Stroke Impact Scale 3.0 [40, 41] Uganda version [42].

*Canadian Occupational Performance Measure* (COPM) measures performance and satisfaction in self-care, productivity and leisure from the client’s perspective using a scale of 1–10. COPM relies on the clients being able to identify their own areas of difficulty [37]. The client is asked 1) to rate performance of the specified activities using a 1 to 10 scale and 2) to score his or her satisfaction with that performance using the same scale. Weighted scores of the chosen activities are added separately for performance and satisfaction to create two summative scores. The summative scores are then divided by the number of rated activities to provide COPM scores that can be used for comparisons across time. A change of two points in the score is seen as a clinically significant change [37].

##### Self-efficacy

The self-efficacy scale is based on social cognitive theory by Bandura [38, 39] and evaluates the individual’s belief in their capability to perform a course of action to attain a desired outcome. The theory of self-efficacy suggests that the stronger the individual’s efficacy expectations, the more likely they will initiate and persist with a given activity. Hence, we hypothesized that participants in the F@ce™ group would have higher self-efficacy and outcome expectations than the CG. To complete the self-efficacy measure, participants were instructed to rate how confident they felt about performing each of 16 everyday activities on a 10-point rating scale ranging from 1) “not confident at all in my ability” to 10) “very confident in my ability”. The self-efficacy scale used was adapted for people with stroke from a similar scale for people with pain [43].

*The Stroke Impact Scale (SIS) 3.0 Uganda version* was used to assess the perceptions of the individual with stroke on functioning in everyday life in eight domains: Strength, Memory and thinking, Emotions, Communication, ADL/ Instrumental activities of daily living (IADL), Mobility, Hand function and Participation. The SIS version 3.0 includes 59 items within these eight domains [41]. Aggregated scores ranges from 0 to 100, the higher the score, the lower the perceived impact of stroke, i.e. fewer problems in everyday life. The SIS 3.0 also includes a question to assess the participant’s global perception of recovery presented in a vertical analogue scale ranging from ‘0 = no recovery to 100 = full recovery’. The SIS 3.0 Uganda version [42] was culturally adapted and psychometrically tested. Changes in the SIS domain scores of approximately 10–15 points appear to represent reasonable definitions of a clinically meaningful change [40]. In the present study, differences in SIS scores <− 14 or < + 14 were categorised as differences of no clinical importance, whereas ≥ –15 or ≥ + 15 were categorised as differences of clinical importance.

### Secondary outcome measures

The *Barthel Index* (BI) [44] was used to assess independence/dependence in ADL. The BI measures independence in 10 self-care and mobility activities. Scores range from 0 to 100, with a lower score indicating greater dependency.

*Occupational Gaps Questionnaire Ugandan version (OGQ-U)* [45] is a self-report measure that facilitates gathering information about, and understanding, the client’s situation with respect to participation in everyday occupations [46]. It measures the extent to which a person performs activities they want to perform, and the extent to which the same person does not perform activities he or she does not want to perform. The OGQ-U is adapted from the English version [47] of the instrument and includes 22 items/activities. On each activity, the respondent is asked two questions; *“Do you perform this activity?”* and *“Do you want to perform this activity?”* If a person responds yes to one of the two questions and no to the other question an occupational gap is noted for that specific activity. Few gaps indicate a better outcome. The OGQ has good validity for different diagnoses, and can be used as a screening tool as it can separate at least two levels of perceived occupational gaps [48]. The median number of occupational gaps in a Ugandan reference sample was 5, which means that in a Ugandan sample > 5 gaps represent restriction in participation in everyday occupations [45].

### Statistical method

Descriptive statistics were used to present the characteristics of the participants and the outcomes. The feasibility of the design regarding patient recruitment, methods and procedures was analysed to determine the potential for realizing a full-scale RCT.

Data were analysed from all participants who completed eight weeks of the study. The comparison of outcomes between the IG and the CG identified whether F@ce™ had any impact on target achievement, self-efficacy, and in perceived impact of stroke, compared to the CG. The outcomes at follow-up, and calculated differences in scores between baseline and follow-up, were compared using the Mann-Whitney U-test for the ordinal data. A *p*-value of 0.05 was accepted as statistically significant. Analyses were conducted using the Statistical Package for the Social Sciences (SPSS).

Ethical approval was granted by the ethical review committee of the Uganda National Council for Science and Technology no. HS 703.

## Results

### Participants and recruitment

**Fig. 1:**
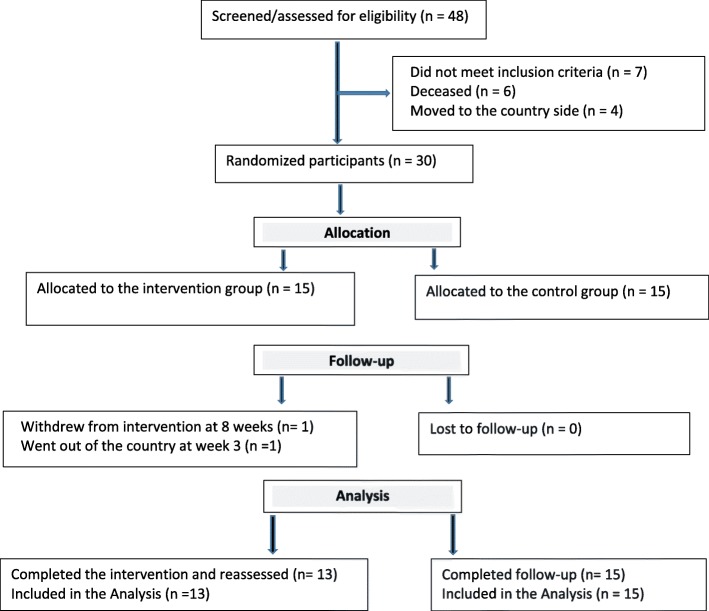
The flow diagram of the study. The recruitment process of participants over the study period

Participants were recruited over a period of one year between March 2016 and February 2017. As shown in the flow chart in Fig. 1, two participants from the IG dropped out, one in the 3rd week because she returned to her home country, and the other in the 8th week for unknown reasons. The IG then comprised *n* = 13 and the CG *n* = 15. The number of participants included from each recruitment site is shown in Table 1. A large number of participants assessed for eligibility in Mulago Hospital neurology ward did not meet the inclusion criteria because most of them came from upcountry homes, and had a mRS score of 5. Six screened tentative participants died before they could be included in the study (Fig. 1). The demographic characteristics of the participants with stroke are shown in Table 2. The IG was older, and had more severe strokes as measured by SSS, than the CG.

**Table 1:** Participants recruited at each site

Site	IG	CG
Referral from Mulago Hospital neurology ward	2	3
Referral from Mulago Physiotherapy outpatient clinic	3	8
Referral from Stroke Rehabilitation Centre -Wampewo	4	2
Referral from Nagulu Hospital outpatient clinic	2	1
Recommended by colleagues	2	1
Total	13	15

**Table 2:** Participants’ demographic characteristics at baseline

	IG *n* = 13	CG *n* = 15
Age, mean (SD)	61.2 (15.0)	58.5 (14.0)
Gender, men/women*, n (%)*	2 (15.4) /11 (84.6)	5 (33.3) /10 (66.7)
Civil status, married/single*, n (%)*	5 (38.5) /8 (61.5)	8 (53.3) /7 (46.7)
Housing, own house/rented house*, n (%)*	13 (100) /0	10 (66.7) /5 (33.3)
Born in Uganda, yes/no*, n (%)*	13 (100)	15 (100)
Level of education, elementary school*/* high school or university, *n* (%)	11 (84.6) /2 (15.4)	10 (66.7) /5 (33.3)
Occupational status before stroke, Employed /occasional work, *n (%)*	3 (23.1) /10 (76.9)	6 (40) /9 (60)
Hemisphere, left/right *n,* (%)	5 (38.5) /8 (61.5)	4 (26.7) /11 (73.7)
Type of Stroke, haemorrhage/ infarction/Unspecified stroke, *n (%)*	3 (23.1) /10 (76.9) /0	3 (20.0) /11 (73.3) /1 (6.7)
Time after stroke, *n (%)*		
3 months – 6 months	10 (76.9)	10 (66.7)
7 months – 11 months	3 (23.1)	3 (20.0)
1 year – 2 years	0	2 (13.3)
Scandinavian Stroke Scale, (0–58), median, (1st, 3rd quartile)	39 (39, 44)	45 (40, 49.3)
Barthel Index,(0–100), median, (1st, 3rd quartiles)	70 (75, 55)	70 (57.5, 95)
Self-efficacy, (0–160) (mean/median)	53 /51	67 /55
Occupational Gaps Questionnaire,(0–22), *Mean (SD)*	14.0 (2.1)	11.5 (2.8)
Canadian Occupational Performance Measure, (0–10), median (1st, 3rd quartile)		
Performance	2.7 (2.3, 3.6)	2.3 (2.0, 4.2)
Satisfaction	2.3 (1.7, 4)	3.0 (2.4, 4.4)
Stroke Impact Scale,(0–100), mean (SD)		
Strength	34.6 (21.1)	40.4 (15.8)
Memory and thinking	69.5 (25.2)	81.4 (19.4)
Emotions	54.3 (11.2)	58.0 (13.0)
Communication	71.2, (28.3)	88.6 (16.2)
Activities of daily living/IADL	41.0 (17.6)	42.7 (19.8)
Mobility	32.3 (22.5)	42.4 (22.3)
Hand function	05.4 (12.8)	10.7 (16.4)
Participation	12.7 (11.8)	19.8 (17.8)
Stroke recovery	46.9 (16.5)	48.0 (09.4)

### Feasibility, fidelity and acceptability

Before the inclusion of participants some semantic adaptations were required to culturally adapt the F@ce™. A core component in the intervention was to set goals, but in the Uganda setting the word targets was used instead. Therefore a semantic adaptation was done. Similarly, the “do” and “check” used in the CADL intervention, were altered to “perform” and “prove” in the F@ce™.

According to the OTs’ logbooks, setting targets by use of the COPM was acceptable and satisfactory for all involved, including the OTs. The targets set by participants in the IG are presented in Table 3. A median number of 13 telephone calls were made for each participant by the OTs, and the most common procedure involved calling both the client and the family member at the same time as the family member was the one that made the training happened. The majority of participants occasionally experienced problems receiving and sending SMS during the eight-week intervention. Whenever the participants did not receive a SMS reminder, due to problems with connection to the telecom operator or for other technical reasons, they wrote their scores down on paper so that they could visually monitor their performance of targets.

**Table 3:** Target activities in F@ce™ during the intervention

	Target 1	Target 2	Target 3
1	Dressing self	Going to the toilet	Communicating with others
2	Dusting windows	Dressing self	Bathing self
3	Bathing self	Washing clothes	Socialization
4	Walk to supermarket	Bathing self	Dressing self
5	Praying activity	Washing clothes	Sweeping
6	Reciting a rosary	Washing clothes	Gardening
7	Washing clothes	Bathing self	Singing common songs with sister
8	Dressing top part of the body	Sorting beans or rice	Grooming
9	Washing clothes	Sorting beans and rice	Visiting (walking to visit)
10	Peeling bananas	Bathing self	Washing clothes
11	Sorting beans outside the house	Making a sign of cross in prayer using the right hand	Feeding using the right hand
12	Feeding	Sorting beans with affected hand	Dressing self, using affected hand
13	Reciting the Rosary during spiritual prayer	Feeding self	Washing clothes

A minimum total of 80 SMS was sent for each participant in the IG group as shown in Table 4.

**Table 4:** Interventions received by the participants during the eight weeks of study

Number of participants who received services	IG *n* = 13	Control *n* = 15
^a^SMS^b^ for eight weeks	11	0
^a^SMS^b^ for six weeks and paper scores for two weeks	2	0
^a^Phone calls from OTs for eight weeks twice a week	13	0
Physiotherapy during the study	8	11
No other rehabilitation during the study	5	4
Hand exercising balls	13	15
Measurement of blood pressure	13	15
Set targets according to COPM^c^	13	15
Received information about stroke and ADL independence	13	15
Assessed at baseline and follow-up	13	15

^a^Included in F@ce intervention (IG)

^b^ *SMS - Short Message Service (text message)*

^c^COPM *Canadian Occupational Performance Measure*

Table 4 also shows the intervention received by both groups, revealing that a large proportion of participants continued to receive other rehabilitation treatments outside the study such as physiotherapy. There was no collaboration between the OTs in the present study and other rehabilitation professionals.

### Primary outcome measures

Table 5 presents the outcomes at baseline and eight-week follow-up, and differences in outcomes between baseline and follow-up for the IG and the CG.

**Table 5:** Primary and secondary outcomes at baseline and follow-up

Measures	Baseline			After 8 weeks			Mean difference		
	F@ce *n* = 13	Control *n* = 15	*P* value	F@ce *n* = 13	Control *n* = 15	*P* value	F@ce *n* = 13	Control *n* = 15	*P* value
Primary outcomes									
COPM performance	2.9	3.4	0.1	5.7	5.8	0.4	2.8	1.6	0.05
COPM satisfaction	3.0	3.3	0.5	5.7	4.9	0.3	2.7	1.6	0.12
Self-efficacy	52.7	67.4	0.8	84.5	81.2	0.4	31.8	13.8	0.04
SIS Strength	34.6	40.4	0.5	50.0	49.1	0.8	15.4	8.7	0.50
Memory	69.5	81.4	0.2	80.2	83.6	0.4	10.1	2.1	0.20
Emotions	54.3	58.0	0.5	60.3	66.3	0.8	5.98	8.3	0.60
Communication	71.2	88.6	0.1	77.2	88.3	0.1	6.04	−0.2	0.65
ADL/IADL	41.0	42.7	1.0	52.1	53.0	0.7	11.1	10.3	0.82
Mobility	32.3	42.4	0.7	45.7	49.8	0.7	13.5	7.4	0.31
Hand function	5.4	10.7	0.2	13.9	20.0	0.4	8.5	9.3	0.96
Participation	12.7	19.8	0.3	22.4	24.0	0.4	9.6	4.2	0.40
Recovery	46.9	48.0	0.4	50.8	58.7	0.2	3.9	10.7	0.48
Secondary outcomes									
Occupational gaps	14.0	11.0	0.6	11.1	11.1	0.1	−2.9	−0.9	0.39
Barthel Index	58.1	72.3	0.2	81.2	80.8	0.9	23.1	8.5	0.06

There was a significant difference between the IG and CG in changes between baseline and follow-up for the primary outcomes COPM performance component and self-efficacy, in favour of F@ce™. The COPM showed that 10 of 13 participants in the IG had a two-point clinically significant improvement in performance compared to 7 of 15 in the CG. In the satisfaction component of COPM, nine participants in the IG had a two-point clinically significant improvement compared to five participants in the CG.

Number of clinically meaningful changes are shown in Table 6, which shows that the IG had a higher number of participants with a 15-point clinically meaningful improvement in six of the eight SIS domains.

**Table 6:** Number of clinically meaningful changes [+ 15 (↑); −15 (↓)] in the Stroke Impact Scale in the groups between baseline and follow-up

SIS domains	IG (*n* = 13)		CG (*n* = 15)	
	↑	↓	↑	↓
Strength	7	1	5	1
Memory	4	0	2	0
Emotions	5	1	2	1
Communication	3	1	1	1
ADL/IADL	6	1	7	1
Mobility	6	1	3	2
Hand function	2	1	3	0
Participation	3	1	2	1
Stroke recovery	4	2	6	1

### Secondary outcome measures

Table 5 presents the mean and the mean differences for all clinical outcomes between baseline and follow-up for both groups. There were no statistically significant differences in outcomes between the IG and the CG for BI (*p* = 0.06) and OGQ *(p* = 0.39), although the IG showed consistent improvement in ADL performance and had fewer occupational gaps than the CG.

## Discussion

To the best of our knowledge, this is the first study in sub-Saharan Africa to evaluate the feasibility of supplying a well-defined family-centred intervention, F@ce™, using mobile phones for persons with stroke and their family members. The primary objectives of this study were to evaluate the feasibility of: i) a mobile phone supported family-centred intervention, F@ce™ and ii) the study design for evaluating the effects of the intervention on the perceived impact of stroke, perceived participation in everyday life and self-efficacy in everyday activities among persons with stroke and their families in Uganda. Overall, the F@ce™ intervention was delivered according to the predetermined design of the intervention, and was found to be viable; it will be feasible in this context with minor alterations. In general, the study design appears feasible, but there are challenges that will need to be addressed in a future full-scale study to evaluate the effects of this mobile phone supported family-centred intervention.

### Feasibility of the intervention

The consent rates as well as the rate of retention of participants throughout the eight-week intervention performs satisfactory. As revealed by the logbooks it is apparent that the interest in receiving the F@ce™ intervention and its content was acceptable among both those who had had a stroke and their family members who turned out to be instrumental for several participants in the study.

The CADL intervention, developed and previously evaluated in a high-income country and from which F@ce™ has been derived, required contextual adaptations to be feasible. This entailed applying a family-centred approach as well as cultural adaptations e.g. adjusting the semantics of the language used and the use of mobile phones with SMS services.

### Family-centred approach

The importance of having a family-centred approach with participation of family members must be emphasised, in realizing training of the person with stroke. Furthermore, the family members appeared to be instrumental in making the training happen. They also established a good working communication with the OTs, and were supportive regarding the SMS communication. It is conceivable that, in this sub-Saharan context, the role of family members [49] has different connotations for people in need of rehabilitation services than the role of family members in high-income countries. In Uganda, the vast majority of the population live in extended families with limited access to affordable health and social services [2, 3]. A large responsibility falls on the family, and a collective approach [50] should therefore be employed in the development and implementation of sustainable community based health services that can reach the majority of people with disability in sub-Saharan Africa [49–51].

### Cultural adaptation of the intervention

To the best of our knowledge, this was the first time the COPM was used in research in Uganda. The instrument was used in setting targets and to monitor performance and satisfaction by the participants - the person with stroke and family members. The activities chosen as targets represented activities that were valued and meaningful, as well as capturing what was perceived as essential activities in everyday life for people in urban Uganda. Previous research [52] has found that goal -setting must be followed by an intervention to achieve a behavioural change, and that change can be sustained even after the intervention has ended. Hence future studies are needed to ascertain that an 8-week intervention render sustainable improvements and behavioural changes.

### F@ce

The F@ce™ intervention was a programme involving the family and the telephone contacts involved talking to the client, the family member or both. Hence it is conceivable that the therapeutic relationship established between the participant, family members and the OT generated a more flexible and sustainable intervention than an individualized intervention would have created. It was assumed that an eight-week time frame for the intervention would be sufficient to achieve the set targets, while at the same time being short enough for participant adherence during the intervention. This was confirmed by the results and the low attrition rate; hence, an eight-week intervention appears feasible. There is a shortage of OTs in Uganda and it is reasonable that the limited resources available should be utilised equally by people in need of rehabilitation services.

As intended, the ability to learn and use the problem-solving strategy “target-plan-perform-prove” appeared to promote self-discovery and adaptation in performing activities. This was expressed as a variation in strategies to achieve the different targets as revealed in the logbooks.

Setting up targets has been reported to require self-interrogation, self-monitoring of performance demands, self-observation and self-evaluation [53]. This is in line with the rationale of the F@ce intervention, which was to enable participants to take agency of their rehabilitation, build a sense of responsibility and to support self-management.

The present finding*s* of improved self-efficacy in the IG, and the ability to learn to use the problem-solving strategy “target-plan-perform-prove” when performing activities is consistent with previous observations, which have shown that a high self-efficacy level is related to good performance in ADL [54]. Hence, we propose that the improvement in self-efficacy at eight weeks in favour of the F@ce™ is likely to be due to the daily practice by the participants in IG and their own monitoring of the training and rating of the own performance.

### Mobile phone support

Use of mobile phones for daily reminders to the participants to perform the target activities, daily reporting of performance ratings, and calls from the OTs twice weekly were feasible. It is conceivable that the daily reminders also promoted the superior positive outcome in self-efficacy in the IG. Scoring the performance of the targeted activities daily may have increased the participant’s awareness of their own performance, and improvements made instilled confidence in their own capacity as has previously been shown in studies on self-management [55]. When the participants occasionally experienced problems sending SMS the performance ratings were written on a paper sheet instead. This might indicate high engagement on the part of the participants and a sign of agency and own responsibility when the ordinary procedures occasionally could not be applied.

Locating the server in Sweden required participants to receive prepaid airtime every day from JTK. The international SMS was expensive and very time-consuming to administer. Hence, to build a similar, sustainable and affordable service a local server should be used, and a local IT technician is essential so that support can be offered immediately.

### Feasibility of the study design

The recruitment of participants was found to be difficult, but the consent rates as well as the rate of retention of participants in the study were good, demonstrating the feasibility of the F@ce™ intervention and the proposed study design in general. The most difficult element was to locate potential participants, since there are no specific rehabilitation units for people with stroke in Uganda. Most stroke patients admitted for acute care on the neurology ward of Mulago hospital either did not meet the inclusion criteria regarding severity of stroke, or could not be contacted after discharge from the hospital. Some were deceased, while others could not be contacted by mobile phone or had travelled to their countryside homes. Furthermore, the recruitment of participants from the Physiotherapy outpatient clinics in the Mulago National Referral Hospital were more reliable than from the private rehabilitation service, and it is possible that the research project encroached on the private service provider’s area of revenue. On the other hand recruiting participants from the physiotherapy department may introduce a selection bias where predominantly participants with physical impairments are included. Therefore in future studies it should be ascertained that also participants with cognitive impairments are included.

This study revealed a number of contextual and economic findings that are relevant for planning a full-powered trial. It is now possible to properly estimate the resource implications of the F@ce™ intervention. During the study, a substantial amount of time was required to complete the assessment protocols both at baseline and follow-up. This led to considerable exhaustion of the participants and the assessor [56]. These experiences could have a measurable effect on the outcomes. It is important that in any future full trial, the number of measurements included in the assessment protocols is decreased. Based on the results of the present study, the recommended outcome measures include the COPM, Self-efficacy scale and the SIS. However, having the assessment protocols installed on a tablet rendered the advantage of no loss of information because it did not allow skipping items, and all data were automatically organised into an excel format and stored securely in the cloud.

### Limitations and future research

This was a pilot feasibility study that was not powered to draw conclusions regarding the effects of the F@ce™ intervention. Caution should thus be exercised when interpreting the results of the effects of the intervention. The sample was small, and the groups were not well balanced. However, it is likely that there are beneficial effects of the F@ce™ intervention because, even though the intervention group was older and had more participants with a severe stroke, it still had the better outcome.

Another limitation is that the majority of the recruited participants in both groups continued to receive physiotherapy during the study. The amount and content of such therapy was not monitored. It cannot be excluded that this therapy might have impacted both the primary and secondary outcomes but in both groups. Even if the CG did not get SMS reminders they probably had some benefits of participating in this study in comparison to most of the people with stroke in Uganda that hardly get any rehabilitation at all.

Another limitation is that participants were only recruited in urban areas. Nevertheless, some of the participants returned to their homes in rural areas and could continue to use the intervention. Further limitations are the lack of: i) allocation concealment, ii) randomization of participants throughout the study, and iii) blinding of the data collector (JTK).

Several issues have been highlighted above that should be addressed in future studies on F@ce™. In addition, the ethics of not supplying rehabilitation services to the control group need to be considered. One method could be to apply a waiting list design [56]. Because about 84% of the population reside in rural area [13], any future research would need to include participants from both urban and rural areas. Furthermore, maintenance of the problem-solving skills needs to be explored by long-term follow-ups.

## Conclusion

The family-centred mobile phone supported F@ce™ intervention will, with some technical adjustments, be feasible for implementation in Uganda. The trial design can be replicated in a larger trial with improvements in recruitment, allocation concealment, randomization and blinding of data collectors. In general, the results support the need for further research in this area, which should also include participants from rural settings.

## Additional file

Additional file 1: Guiding principles for implementation of the mobile phone supported F@ce™. A description of the used guiding principles used by the OTs during the implementation of the intervention. (DOCX 17 kb)
