# Supplementary material for: A feasibility study of a mobile phone supported family-centred ADL intervention, F@ce™, after stroke in Uganda
Source: Global Health. 2018 Aug 15;14:82. doi: 10.1186/s12992-018-0400-7 (PMC6094578; doi:10.1186/s12992-018-0400-7)
Supplement: Supplementary file 1 — Guiding principles for implementation of the mobile phone supported F@ce™. A description of the used guiding principles used by the OTs during the implementation of the intervention. (DOCX 17 kb) [file 12992_2018_400_MOESM1_ESM.docx]

**Additional file 1: Appendix 1. Guiding principles for implementation of the mobile phone supported F@CE^TM^**

| **Principle** | **How it was implemented and encouraged** |
| --- | --- |
| ***Basic knowledge about stroke and rehabilitation*** | To explore what the client and the family member knew about stroke, what they wanted to know and any concerns that they felt might hamper rehabilitation. Information about prevention of another stroke and regular check-ups of predisposing conditions such as hypertension, diabetes, etc. |
| ***Applying a Phenomenological perspective*** | To take the point of departure in the client’s life world, understand the client’s experiences and the clients’ own everyday life in relation to what they wanted and needed to do. |
| ***Applying a family-centred perspective*** | A family-centred perspective entailed an invitation to the client to involve a family member to be an active person in planning, negotiation and support in the 8-weeks of intervention to fit into the family everyday life. |
| ***Explaining the term Problem solving strategy*** | To introduce the client to use a problem-solving strategy where TARGETs mean to set three goals in activities the person want and need to do. Followed by a PLAN with actual strategies that should be used during the practice of the activities during 8 weeks (=PERFORM). PROVE include the follow up calls and assessments. |
| ***Setting of targets in a family-centred approach*** | To encourage the client to think of the most valued activities at home that they missed doing. The activities that made the client feel more independent and valued. The family member was involved in the dialogue. Unrealistic targets were modified and the client was guided to formulate achievable and measurable targets. |
| ***Encouraging self-discovery and self- management Strategy*** | To ask the client on how three practiced activities were managed during assessment and setting of targets. This encouraged the client to exploit undiscovered capacities that needed to be explored in order to meet the encountered challenges. The client was encouraged to maintain previously acquired praxis and to make new innovative strategies. |
| ***Involving family members in the SMS-service*** | Family members were given information about the importance of independent performance of activities by the client in order to be supportive and encouraging in the performance of activities. When the client could not manage the mobile phone, the family member received the SMS, supported the client to rate the performance, send scores by SMS and encouraged the client to perform the activities. |
| ***Client´ reflecting on previous experiences*** | To encourage the client to reflect on strategies used before, while performing similar activities. This was to assist the client to use previously learned skills and adapt them to meet new related situations. |
